# Impaired Fear Extinction as Displayed by Serotonin Transporter Knockout Rats Housed in Open Cages Is Disrupted by IVC Cage Housing

**DOI:** 10.1371/journal.pone.0091472

**Published:** 2014-03-21

**Authors:** Ling Shan, Pieter Schipper, Lourens J. P. Nonkes, Judith R. Homberg

**Affiliations:** Donders Institute for Brain, Cognition, and Behaviour, Centre for Neuroscience, Department of Cognitive Neuroscience, Radboud University Nijmegen, Medical Centre, Nijmegen, The Netherlands; INSERM/CNRS, France

## Abstract

Anxiety disorders are influenced by both environmental and genetic factors. A well-known example for gene x environment interactions in psychiatry is the low activity (s) allelic variant of the serotonin transporter (5-HTT) promoter polymorphism (5-HTTLPR) that in the context of stress increases risk for depression and post-traumatic stress disorder (PTSD). Previously, we observed robust anxiety-related phenotypes, such as an impairment in fear extinction, in 5-HTT knockout (5-HTT^−/−^) versus wild-type (5-HTT^+/+^) rats housed in open cages. Recently, housing conditions were changed from open cages to individually ventilated cages (IVC), which are associated with a high ventilation fold and noise. This switch in housing conditions prompted an unplanned 5-HTT gene x environment interaction study in our rats. The current study shows that lifetime stress by means of IVC cage housing abolished genotype differences in fear extinction between 5-HTT^−/−^ and 5-HTT^+/+^ rats. Although this effect was not attributed specifically to either the 5-HTT^+/+^ or the 5-HTT^−/−^ genotype, the findings are in agreement with the modulatory role of serotonin in the processing of environmental stimuli. Our findings also underline the possibility that housing conditions confound the interpretation of anxiety-related behaviours in rodents.

## Introduction

A large body of evidence has shown that the laboratory environment and rearing conditions can have a huge impact on brain development and influence the outcome of behavioural tests [1]. Housing is of special interest as it has a consistent influence on behavioural phenotyping across different experimenters or locations [2]. Anxiety-related phenotypes are especially sensitive to environmental influences compared to other stable phenotypes, such as ethanol preference and locomotion activity [3], [4], [5]. Accordingly, Wahlsten et al. have noted behavioural differences in the elevated plus maze test for anxiety upon moving a laboratory within an university campus [5].

Our lab extensively works with the serotonin transporter (5-HTT) knockout rat model [6] for understanding gene x environment interactions [7]. These 5-HTT^−/−^ rats exhibit anxiety- and depression-like symptoms that resemble those seen in stressed human subjects carrying the short (s) allelic variant of the 5-HTT linked polymorphic region (5-HTTLPR) [8]. The 5-HTTLPR s/s genotype is associated with heightened fear and anxiety, risk for post-traumatic stress disorder (PTSD) [9] and major depression following stress [10]. The underlying behavioural mechanism for trait anxiety and risk for depression in the context of environmental stressors is thought to be related to hypervigilance, as both the 5-HTT^−/−^ rats and 5-HTTLPR s-allele carriers are highly sensitive to aversive environmental stimuli [11].

One particular robust phenotype demonstrated by 5-HTT^−/−^ rats is an impairment in fear extinction [12]. During extinction, when the fear-associated stimulus is presented in the absence of the aversive outcome (i.e. footshock), 5-HTT^−/−^ rats show remarkably higher levels of freezing compared to 5-HTT^+/+^ rats [12]. A deficit in the ability to acquire or retain extinction memory is a critical feature of PTSD (American Psychiatric Association, 1994).

5-HTT experimental data has shown that both in early life and adulthood stress interacts with 5-HTT genetic variance [7]. As an example of the numerous studies that have been reported: when pregnant and lactating mice were exposed to soiled bedding of unfamiliar males - which is hypothesized to be dangerous environment - offspring exhibited enhanced anxiety-like behaviour in the elevated plus maze and dark-light box tests, and reduced exploratory locomotion in the open field compared to offspring of dams that were exposed to neutral bedding. The effects were most pronounced in 5-HTT^−/−^ mice as compared to 5-HTT^+/−^ or 5-HTT^+/+^ mice [13]. Furthermore, postnatal, footshock exposure suppressed exploratory behaviour and increased anxiety-like behaviour in the light-dark box, elevated plus-maze and open field tests, as well as increased depression-related behaviour following repeated exposure to forced swim stress in 5-HTT^−/−^, but not 5-HTT^+/+^, mice [14]. Not only stress in early life, but also stress in later life differentially effects 5-HTT^−/−^ and 5-HTT^+/+^ rodents. For example, 5-HTT^−/−^ mice exhibited longer escape latencies in a gene-dose dependent manner (5-HTT^−/−^>5-HTT^+/−^>5-HTT^+/+^) after repeated inescapable footshock stress exposure, whereas there were no such genotype differences under stress free conditions [15]. Using social defeat as environmental factor in adult mice, adult 5-HTT^−/−^ mice that lost during aggressive encounters showed more anxiety-like behaviour in the elevated plus maze test and less exploration compared to 5-HTT^+/+^ mice [16]. Additionally, socially defeated 5-HTT^−/−^ mice exhibited delayed fear extinction, which was not seen among non-socially defeated mice [17]. Overall, these data indicate that 5-HTT^−/−^ rodents show increased anxiety- and depression-related behaviour after a history of stress experiences regardless as to whether the stress takes place in early life or adulthood. However, when 5-HTT^−/−^ and 5-HTT^+/+^ rats from prenatal development until testing were exposed to construction work in the animal facility - which is associated with extreme noise and vibrations - we observed that 5-HTT^−/−^ anxiety-related phenotypes in the elevated plus maze and social interaction tests were abolished. This was due to increased anxiety in 5-HTT^+/+^ rats and decreased anxiety in 5-HTT^−/−^ rats [18]. Hence, if stressors are too severe, the specific modulation of phenotypes by the 5-HTT^−/−^ genotype may be lost.

Recently, housing conditions were changed, which prompted an unplanned 5-HTT gene x environment interaction study in our rats. The major change in housing involved a switch from housing in open cages to housing in individually ventilated cages (IVC). These cages are characterized by a high ventilation-fold (maximal air speed 0.05 m/s), which the animals may experience as windy. Furthermore, to generate this high ventilation-fold, an attached motor for Easy & Smart flow generated constant noise (63 dB). Given that IVC housing is associated with stress levels that may be as severe as that of construction noise, we hypothesized that IVC cages housing leads to normalization of the impaired fear extinction in 5-HTT^−/−^ rats by affecting neither the 5-HTT^−/−^ rats nor the 5-HTT^+/+^ rats specifically.

## Methods

Serotonin transporter knock-out rats (Slc6a4^1Hubr^) were generated by ENU-induced mutagenesis [6]. Experimental animals were derived from crossing 4–8 month old homozygous 5-HTT^−/−^ rats that were out-crossed with commercial (Harlan, Ter Horst, The Netherlands) wild-type Wistar 5-HTT^+/+^ rats for at least 10 generations. Ear cuts for genotyping were taken after weaning at the age of 21 days. The rats were aged between 12 and 20 weeks.

Two group of animals (ten 5-HTT^−/−^ *versus* ten 5-HTT^+/+^ and five 5-HTT^−/−^ *versus* five 5-HTT^+/+^ rats) were bred and housed in standard Macrolon type 3 cages (42×26×20 cm). Another two group of animals (seven 5-HTT^−/−^ *versus* eight 5-HTT^+/+^ and five 5-HTT^−/−^ *versus* five 5-HTT^+/+^ rats) were bred and housed in Sealsafe Plus green line IVC GR900 cages (39.5×34.6×21.3 cm). All other factors are identical for all rats. That is, all animals were housed in a temperature (21±1°C) and humidity (45–60% relative humidity) controlled room, on a 12/12 h light-dark cycle, with *ad libitum* access to water and food until testing. Three-month-old male rats were used for the experiments.

The first fear extinction experiment – conducted by LN - was based on ten 5-HTT^−/−^ and ten 5-HTT^+/+^ rats housed in open cages. This experiment was replicated by P.S. using an independent group of rats consisting of five 5-HTT^−/−^ and five 5-HTT^+/+^ rats. The same fear conditioning and extinction paradigm was applied by P.S. in a group of seven 5-HTT^−/−^ and eight 5-HTT^+/+^ rats that were housed in IVC cages from birth until testing, as well as in an additional independent group of five 5-HTT^−/−^ and five 5-HTT^+/+^ IVC cages housed rats.

All experiments were approved by the Committee for Animal Experiments of the Radboud University Nijmegen Medical Centre, Nijmegen, The Netherlands, and all efforts were made to minimize animal suffering and to reduce the number of animals used (DEC number 2012-271 and 2011-234). The animals were all habituated to the conditioning chamber the day prior to conditioning for 10 minutes. Starting three days prior to the start of the experiment, the animals have been handled three times daily for 1–2 minutes each time.

### Fear conditioning

The first fear extinction experiment (open cages, N = 10/genotype) was performed using two modified Skinner boxes (TSE Systems GmBH, Bad Homburg, Germany), which have been described in detail previously [12]. For all other experiments, Med Associates Inc. conditioning boxes were used for fear conditioning. The experimental protocol used was identical for all groups. Thus, the animals were placed in the conditioning boxes for 10 minutes, during which they were subjected to a tone (CS) five times followed by a footshock (US) with an 1 minute interval and 2 minutes habituation. Before each session of fear conditioning the conditioning boxes were cleaned and the program was re-calibrated. Animal behaviour was recorded.

### Fear extinction

Fear extinction was performed in a different experimental room than the room where fear conditioning took place. After an habituation period of 2 min, the animals received the CS 24 times without shock in a period of 15 minutes. The extinction arena was cleaned before each session of fear extinction. Animal behaviour was recorded for later analysis.

### Scoring

Conditioned freezing of the rats was manually scored using homemade behavioural observation software. Behaviour was scored manually, and the behavioural software provided event-logging functionality, similar to “Noldus Observer”. The observers were blind to subject genotype and housing conditions. Freezing behaviour was defined as complete lack of movement except for the muscle movements needed for respiration.

### Statistical analysis

Statistical analyses were carried out using SPSS Statistics 21.0 (SPSS Inc, Chicago, IL). In order to be normally distributed, the log10 transformation was applied on the original data. To analyze pre-CS freezing between two groups independent t-tests were used, and analysis of pre-CS freezing between four groups (2 genotypes, two housing conditions) was performed using two-way ANOVA. CS-induced freezing was analyzed in trial blocks of 3 CSs using a two- or three-way (repeated measures) ANOVA. P values lower than 0.05 were considered as statistical significant. Values are presented as mean ± standard error of the mean (SEM).

## Results

**Figure 1:**
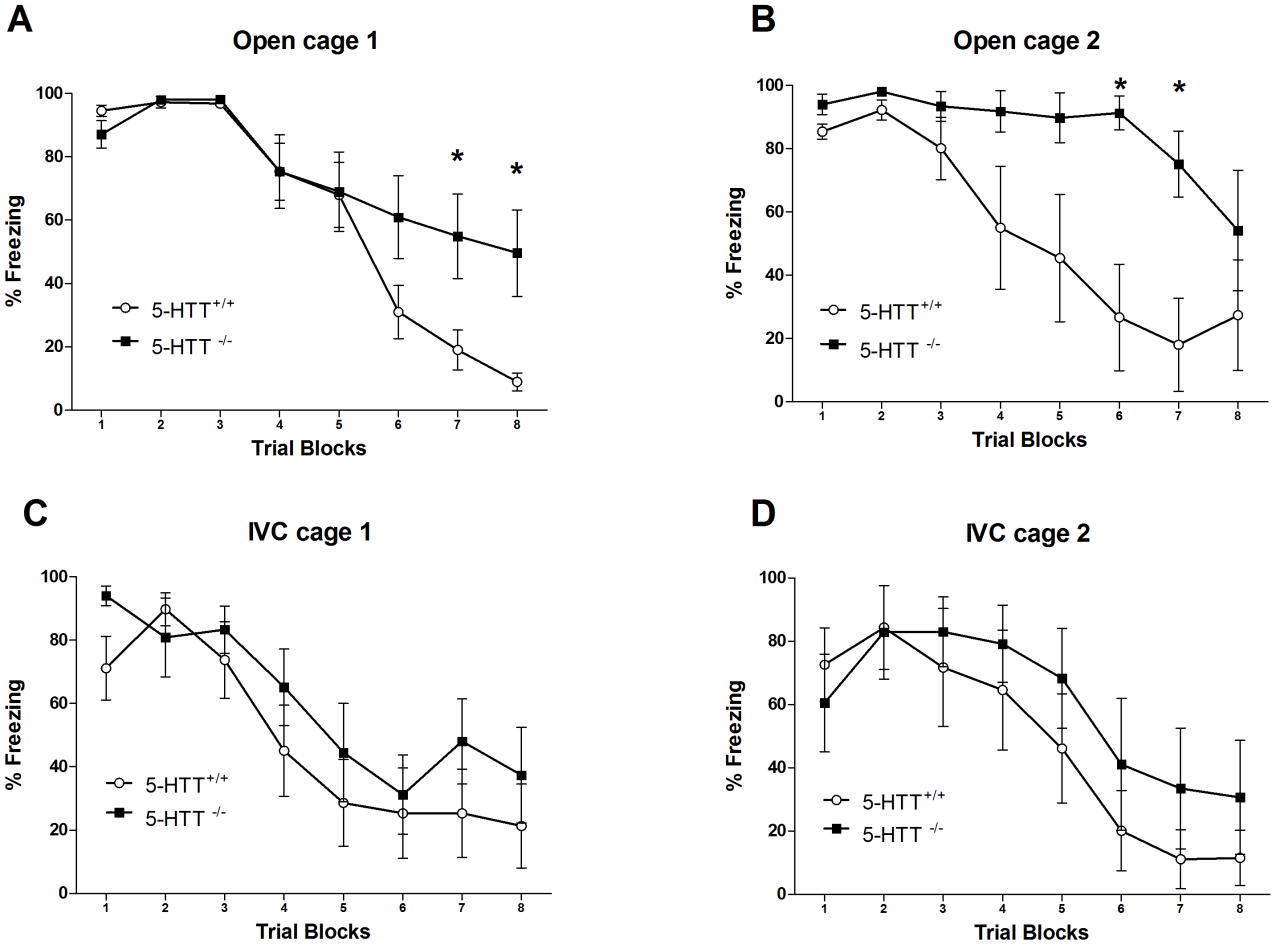
Conditioned freezing behaviour as observed during the fear extinction session conducted 24 hours after fear conditioning. Freezing behaviour was impaired in 5-HTT^−/−^ (n = 10) compared to 5-HTT^+/+^ (n = 10) rats housed in open cages (A); The replication experiment also showed impaired fear extinction in 5-HTT^−/−^ (n = 5) *versus* 5-HTT^+/+^ (n = 5) rats housed in open cages (B); Lack of differences in freezing behaviour across trials between 5-HTT^−/−^ (n = 7) and 5-HTT^+/+^ (n = 8) rats housed in IVC cages (C); This lack of differences in freezing behaviour was confirmed in an independent replication experiment consisting of five 5-HTT^−/−^ and five 5-HTT^+/+^ rats housed in IVC cages (D). Data (trial blocks averaged over 3 trials) represent mean percentage of freezing time (± S.E.M.) during CS presentation. * P<0.05.

Using ten 5-HTT^−/−^ and ten 5-HTT^+/+^ rats housed in open cages, no genotype differences in pre-CS freezing were observed (0.000±0.000 vs 0.059±0.059, t = −1.000, P = 0.331, NS, independent t-test). In contrast, a significant genotype x trial-block interaction was observed for CS-induced freezing (F_(7,126)_ = 4.709, P<0.01), without a main genotype effect (F_(7,126)_ = 1.390, NS). Subsequent analysis of the genotype x trial-block interaction indicated that 5-HTT^−/−^ rats showed significantly higher levels of freezing behaviour during trial blocks seven (F_(1,18)_ = 4.445, P<0.05) and eight (F_(1,18)_ = 5.545, P<0.05), compared to their wild-type counterparts (Figure 1A).

Importantly, in a replication experiment conducted under similar conditions (including open cages) using another five 5-HTT^−/−^ and five 5-HTT^+/+^ rats, we obtained similar results. Thus, a significant genotype x trial-block interaction was observed for CS-induced freezing (F_(7,56)_ = 4.553, P<0.01), with a main genotype effect (F_(1,8)_ = 6.298, P<0.05). Moreover, the genotype x trial-block interaction indicated that 5-HTT^−/−^ rats showed significantly higher levels of freezing behaviour during trial-blocks six (F_(1,8)_ = 7.834, P<0.05) and seven (F_(1,8)_ = 11.969, P<0.01) (Figure 1B). Pre-CS freezing could not be analyzed, because it was close to 0.

When 5-HTT^−/−^ and 5-HTT^+/+^ rats were housed in IVC cages, we observed that pre-CS freezing was higher in seven 5-HTT^−/−^ than eight 5-HTT^+/+^ rats (26.231±9.053 vs 8.348±5.637, t = −2.466, P<0.05, independent t-test). However, under these stressful housing conditions no genotype x trial-block interaction (F_(7,91)_ = 1.629, NS), nor a genotype effect (F_(7,91)_ = 3.504, NS) was observed for CS-induced freezing (Figure 1C).

When we replicated the experiment in an independent group of IVC housed animals, again no genotype effects were observed (Figure 1D). The pre-CS freezing was not different between the five 5-HTT^+/+^ and five 5-HTT^−/−^ animals (3.268±1.294 vs 13.535±5.839, t = −1.620, P = 0.144, NS, independent t-test). Additionally, there was no genotype x trial-block interaction (F_(7,56)_ = 0.692, NS), nor a genotype effect (F_(1,8)_ = 0.690, NS).

**Figure 2:**
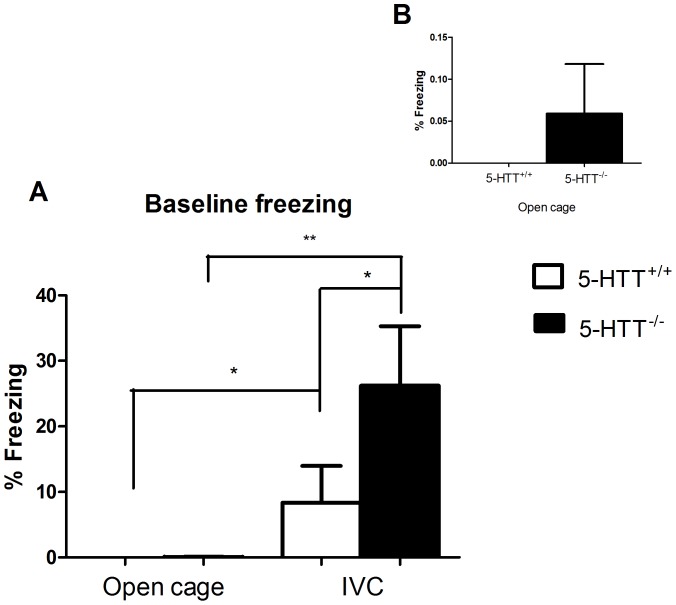
Pre-CS conditioned freezing behaviour is significantly higher in 5-HTT^−/−^ rats compared to 5-HTT^+/+^ rats regardless of housing conditions. (A) The inset figure shows increased pre-CS freezing in 5-HTT^−/−^ rats compared to 5-HTT^+/+^rats housed in open cages (B). Data represent mean ± S.E.M. * P<0.05, ** P<0.01.

When combing the data derived from open cage and IVC cage housed rats for the pre-CS freezing values we found a significant (F_(1,31)_ = 7.726, P<0.001) genotype×cage effect as well as significant genotype (F(_1,31)_ = 8.719, P<0.01), cage (F_(1,31)_ = 39.073, P<0.001) effects, respectively (the replication batches were not included, see above) (Figure 2). Subsequently, using an independent t-test we compared the pre-CS freezing behaviour of animals housed in open cages *versus* animals housed in IVC cages, and found that pre-CS freezing was significantly higher in rats housed IVC cages compared to rats housed in open cages, regardless of genotype. That is, increased pre-CS freezing was observed in both 5-HTT^+/+^ (8.348±5.637 vs 0.000±0.000, t = −2.192, P<0.05) and 5-HTT^−/−^ (26.231±9.053 vs 0.059±0.059, t = −7.625, P<0.001) rats housed in IVC cages versus open cages. Furthermore, the 5-HTT^−/−^ animals showed significantly higher pre-CS freezing (26.231±9.053 vs 8.348±5.637, t = −2.466, P<0.05) compared to 5-HTT^+/+^ rats when housed in IVC cages, but not when housed in open cages (0.000±0.000 vs 0.059±0.059, t = −1.000, P = 0.331, NS) (Figure 2).

**Figure 3:**
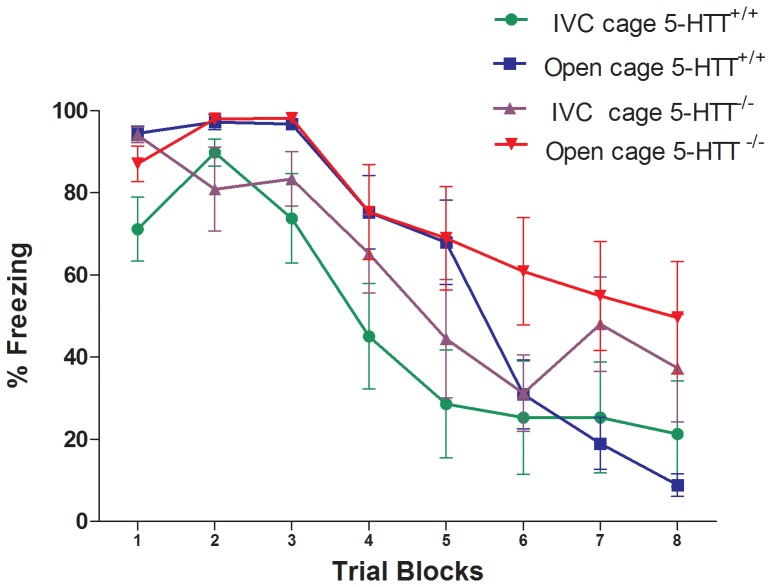
Lack of differences in conditioned freezing behaviour between the first experiment of 5-HTT^−/−^ rats housed in open cages (red), the first experiment of 5-HTT^−/−^ rats housed in IVC (purple), the first experiment of 5-HTT^+/+^ rats housed in open cages (blue), and the first experiment of 5-HTT^+/+^ rats housed in IVC (green). Data (trial blocks averaged over 3 trials) represent mean percentage of freezing time (± S.E.M.) during CS presentation.

When comparing CS-induced freezing among the IVC cages versus open cage housed animals (without replications) we found no trial-block x genotype x cage interaction (F_(7, 217)_ = 0.731, P = 0.646, NS). In addition, there was no cage x genotype interaction (F_(1, 31)_ = 0.939, P = 0.340, NS) (Figure 3). However, there was a genotype effect (F_(1,31)_ = 5.331, P<0.05), and the cage effect was close to significance (F_(1,31)_ = 3.577, P = 0.068) (Figure 3). These data indicate that fear extinction was overall lower in 5-HTT^−/−^ compared to 5-HTT^+/+^ rats, regardless of cage type.

## Discussion

The current study demonstrates that housing conditions can have a paramount effect on conditioned freezing behaviour in rats, as function of 5-HTT genotype. Specifically, we found that the impaired fear extinction in 5-HTT^−/−^ rats was abolished when the animals were bred and housed in IVC cages instead of open cages. Because the standard operating procedures, bedding materials, food, room temperature and humidity were identical for the open and IVC cages housed animals, we believe that differences in the caging systems and the associated ambient noise levels most likely contributed to the abolishment of genotype differences in fear extinction.

Both 5-HTT^−/−^ and 5-HTT^+/+^ rats housed in IVC cages showed significantly longer pre-CS freezing behaviour than rats housed in open cages. This implies that the IVC cage-related noise and ventilation increased stress levels regardless of 5-HTT genotype. Yet, generally, pre- and postnatally stressed 5-HTT^−/−^ rodents are more sensitive to stressful stimuli compared to 5-HTT^+/+^ rats [7]. Possibly, 5-HTT genotype only modulates stress effects if the stress is moderate. When the stress is severe, this 5-HTT genotype specific modulation may be lost, as we observed in the current study as a consequence of IVC cages housing stress. In support, unpredictable chronic mild stress reduced anxiety-like behaviour in adult male 5-HTT^−/−^ mice, making them indistinguishable from 5-HTT^+/+^ mice [19]. Our findings are also in line with a previous report showing that independent from genetic background IVC cages housed rodents exhibited increased c-Fos expression in the paraventricular nucleus of hypothalamus [1], which plays a key role in hypothalamic-pituitary-adrenal axis-mediated stress responses [20]. Additionally, we showed that construction noise stress abolished genotype differences in behaviour as measured in the elevated plus maze and social interaction tests, in both 5-HTT^+/+^ and 5-HTT^−/−^ rats [18]. Whereas these observations were made in male rats, Joeyen-Waldorf et.al. reported that female 5-HTT^−/−^ rats exhibited a normal non-stressed baseline, but highest chronic mild stress-induced emotionality [19]. As to whether the IVC cage-induced stress is also modulated by sex deserves further study.

Some limitations have to be noted. First of all, evidence suggests that particularly 5-HTT^+/−^ rodents are most sensitive to environmental influences, compared to 5-HTT^−/−^ and 5-HTT^+/+^ rats [7], [21]. Although 5-HTT^+/−^ rats do not differ from 5-HTT^+/−^ rats regarding fear extinction (Schipper, Homberg, unpublished data), IVC stress could interact with the 5-HTT^+/−^ genotype. Therefore, it is unfortunate that we did not include the heterozygous animals in this ‘unplanned’ study. Another limitation of this study is that the number of animals tested under IVC cages housing conditions was rather low (n = 5). However, five rats per group were sufficient to obtain significant genotype effects among the open cage housed rats, suggesting that we did not encounter a lack of power. Because the current gene x environment interaction study was unplanned, we do not have further data available on the brain stress system to understand how IVC cages stress abolishes genotype effects in rats.

In sum, the current study shows that lifetime stress by means of IVC cages housing abolished genotype differences in fear extinction between 5-HTT^−/−^ and 5-HTT^+/+^ rats. Although this effect was not attributed specifically to either the 5-HTT^+/+^ or the 5-HTT^−/−^ genotype, the findings are in agreement with the modulatory role of serotonin in the processing of environmental stimuli [11]. Given that housing conditions are typically not considered as experimental factors, our findings may increase awareness of the possibility that housing conditions can confound the interpretation of anxiety-related behaviours in rodents.

## Supporting Information

Table S1 **Row data.**
(XLSX)
